# Breast Edema in Women With Arm Lymphedema Following Breast-Conserving Surgery: A Systematic Review

**DOI:** 10.7759/cureus.108129

**Published:** 2026-05-02

**Authors:** Pierina S Barletti, Megan T Moldovan, Maliha Latif, Shanelle Bryan, Grace Blair, Harvey N Mayrovitz

**Affiliations:** 1 Medical School, Nova Southeastern University Dr. Kiran C. Patel College of Osteopathic Medicine, Davie, USA; 2 Medical Education, Nova Southeastern University Dr. Kiran C. Patel College of Allopathic Medicine, Davie, USA

**Keywords:** arm lymphedema, axillary lymph node dissection, breast cancer, breast-conserving surgery, breast edema

## Abstract

Breast edema is a clinically significant but underrecognized complication following breast-conserving surgery (BCS) and radiotherapy. It involves parenchymal and cutaneous changes that can cause pain, functional impairment, and reduced quality of life. Despite overlapping risk factors and shared lymphatic pathophysiology with breast cancer-related lymphedema (BCRL), breast edema remains poorly defined, with no standardized diagnostic criteria and wide variability in reported incidence. In contrast to the extensive literature on arm lymphedema, breast edema, particularly in patients who develop BCRL, has not been systematically synthesized. A clearer understanding of this relationship is needed to improve recognition, diagnosis, and clinical management. This systematic review aims to analyze the association between breast edema and BCRL, focusing on the development of breast edema and its quantitative diagnostic criteria following BCS. Three databases (Ovid MEDLINE, EMBASE, and Web of Science) were searched for studies pertaining to breast edema and arm lymphedema following BCS. Studies underwent a multistep screening process to ensure they met the inclusion criteria before inclusion in this systematic review. To be included in this review, studies must have been peer-reviewed, published between 2015 and 2025, written in the English language, and relevant to the topic of breast cancer-related lymphedema following BCS. A total of 23 studies met our inclusion criteria, representing 10,402 women across 14 countries who underwent BCS. The prevalence of breast edema varied widely, ranging from 2.7% to 24.8% in clinically assessed cohorts and up to 72% in ultrasound (US)-based studies. Breast edema was more frequently observed following axillary lymph node dissection, larger breast volume, higher body mass index (BMI), postoperative cellulitis, and in patients with BCRL. Women with breast edema reported worse quality of life, greater breast pain, and poorer body image. Quantitative assessment methods, including US and tissue dielectric constant (TDC) measurements, identified both clinical and subclinical breast tissue changes, though diagnostic thresholds and assessment approaches varied substantially across studies. Overall, this review illustrates multiple factors that contribute to breast edema and arm lymphedema, yet also points to the need to improve assessment measures to both identify and prevent edema following BCS.

## Introduction and background

Breast edema is a multifaceted condition that encompasses more than the distinctive swelling of the breast. Other commonly reported criteria in literature include peau d'orange, a feeling of breast heaviness, skin thickening, breast pain, redness, hyperpigmented pores, and a positive pitting sign [1]. Breast edema has also been observed to be composed of two components [2,3]. The first is a generalized enlargement or swelling of the breast tissue, referred to as "parenchymal edema" [2]. The second component involves edematous changes in the epidermis and dermis, referred to as "cutaneous edema" [2]. Although cutaneous edema can occur independently, the two components most often co-occur [2]. Breast edema may also be characterized by increased skin thickness and breast parenchymal density, accompanied by pronounced interstitial markings [3]. Although there is a general consensus on the common features of breast edema, a standardized set of diagnostic criteria for its definition is lacking in clinical practice, hindering its consistent diagnosis and assessment. Breast edema can be painful and impact a patient psychologically, socially, personally, physically, and professionally, making it essential for clinicians and researchers to understand and recognize this condition [4].

Several risk factors have been previously linked to the development of breast and arm lymphedema after treating breast cancer. These include the extent of surgical intervention, intensity of radiation treatment, initial presence of inflammatory breast carcinoma, postoperative infection, larger breast and tumor size, obesity, and diabetes [3-5]. Earlier studies also suggested that a shorter interval between breast surgery and the initiation of radiotherapy predisposes patients to developing breast edema [6,7]. In one study, significant predictors of breast edema were the extent of axillary surgical intervention, presence of arm lymphedema, higher body mass index (BMI), and tumor grade [8]. Although the findings were consistent regarding the role of axillary intervention and tumor characteristics, this study did not find significant associations with inflammatory breast carcinoma or postoperative infection, likely due to the small sample size of 105 patients with breast cancer [8].

Postoperative radiotherapy is the standard of care after breast-conserving surgery (BCS), as it provides oncologic outcomes comparable to mastectomy [9]. The development of breast edema following this treatment is a significant concern [8,10]. Breast edema may also result from less common causes such as inflammatory breast carcinoma, congestive heart failure, mastitis, lymphatic obstruction (from axillary, chest wall, or intrathoracic lesions), metastasis, breast lymphoma, or trauma [1,3]. Impairment of lymphatic function, due to combined BCS and radiotherapy, may cause significant breast edema [1]. This effect is expected, given the importance of lymphatic drainage in cancer metastasis and its alteration after breast treatment or axillary lymph node removal [11]. In addition to fluid buildup, tissue changes, such as fat accumulation and fibrosis, can alter the extracellular matrix and worsen lymphatic dysfunction [1,12]. Understanding the causes and tissue changes associated with breast edema underscores the importance of accurately measuring breast edema for proper evaluation and management.

Breast edema incidence can be assessed using both quantitative and qualitative methods, though options remain limited. Traditional diagnosis and staging of breast edema primarily rely on physical examination, which involves observation and palpation [11,13]. Early use of patient questionnaires was insufficiently specific [14] and was followed by the development of the Breast Edema Questionnaire (BrEQ), the first validated self-reported questionnaire for assessing breast edema in patients with breast cancer treated with BCS and radiation therapy [15]. The BrEQ was reported to be able to distinguish between patients with and without breast edema and included additional parameters, such as correlating thicker skin with higher symptom scores. Although studies on the cultural adaptation of the BrEQ are lacking, its ease of use may support early detection, timely referral, and potential application in both clinical practice and research.

Quantitative breast edema assessment methods include breast ultrasound (US), which measures dermal thickness to provide continuous evaluation of cutaneous edema, offering an accessible, non-invasive approach [2,16]. However, its measurements may not always correlate with changes in the volume and symptoms women perceive [2,16,17]. Therefore, it is important to capture serial US measurements of dermal thickness and assessments of symptoms experienced by women [2,17]. Tissue dielectric constant (TDC) is another non-invasive, rapid 10-second method that measures local tissue water content [18,19]. Studies have shown consistently high TDC values in the operated breast compared to the healthy breast, even before radiotherapy, stressing the impact of surgery on breast edema [20]. In addition, reductions in TDC have been observed with the use of compression bras, correlating with patients’ reported improvement in breast heaviness [21]. The utility of TDC in routine clinical practice remains undetermined.

The reported incidence of breast edema related to breast cancer treatment varies widely, ranging from 10% to 90.4% [5,14]. Conservative and noninvasive treatments, such as skin care, manual lymphatic drainage, taping of the breast, exercise therapy, and compression, aim to provide long-term intervention that prevents worsening of breast edema [1,4,5]. However, evidence for the efficacy of these treatments is scarce [1,4]. Moreover, out-of-pocket costs for these treatments can also be substantial and contribute to non-compliance [21].

However, these treatments are recommended to reduce the risk of cellulitis, a common and potentially serious complication of breast edema as well as breast cancer-related lymphedema (BCRL) [1,22,23]. BCRL is characterized by swelling of the upper extremities due to the accumulation of interstitial fluid and typically occurs on the affected side of the body, although it may also occur on the unaffected side in some cases [24]. In contrast to breast edema, which in some cases may resolve over time, BCRL is a chronic and progressive condition if not treated [25,1]. Despite these distinctions, both breast edema and BCRL share the same key risk factors, including surgical intervention, treatment with radiation, presence of inflammatory breast carcinoma, postoperative infection, and higher BMI [8]. Specifically, surgeries such as axillary lymph node dissection or sentinel node biopsy can affect lymphatic pathways in both the breast and the arm, which may lead to breast edema and arm lymphedema, respectively [1,5]. From a pathophysiological perspective, both conditions result from impaired lymphatic drainage and accumulation of protein-rich interstitial fluid. This is then followed by fat deposition, fibrosis, and increased risk of skin infections [26]. Importantly, although arm lymphedema has been extensively studied, breast edema has been comparatively underexplored. This may be due to the lack of validated assessment tools and a lack of consensus on diagnostic criteria [27]. A starting point for developing targeted, evidence-based interventions is further understanding the risk factors and prevalence of both conditions to benefit patients through education and preventive and curative therapies [5]. Therefore, lymphedema prevention strategies should address both breast and arm manifestations after breast cancer therapy [21].

Systematic summarization of what is known about the development of breast edema associated with BCRL has not been previously addressed. This review aims to fill this gap by addressing what is known about breast edema in the context of BCRL, including current diagnostic methods and the correlation between lymphedema of the arms and breasts following breast-conserving treatment for breast cancer.

## Review

Methods

Study Design

This systematic review was conducted in accordance with the Preferred Reporting Items of Systematic Reviews and Meta-Analyses (PRISMA) 2020 guidelines [28]. The research question was designed to critically investigate what is known about the development of breast edema in women, with the following specific research question being addressed. In women who develop arm lymphedema after being treated with BCS for breast cancer, what is known about the development of breast edema? Study selection was done with pre-defined criteria according to the Population, Intervention, Comparator, Outcomes (PICO) framework [29], with the following components: Population = women with breast cancer-related arm lymphedema, Intervention = examination of the intersectionality between BCRL and breast edema, Comparison = women who develop BCRL without breast edema, and Outcome = correlation between BCRL and breast edema due to treatment methods, risk factors or other undetermined reasons.

Eligibility Criteria

Studies were eligible for inclusion if they involved women over the age of 18 years diagnosed with breast cancer who developed BCRL following BCS. The focus was on studies that examined the development of breast edema, including its incidence, clinical features, and relationship to arm lymphedema. Eligible sources were peer-reviewed articles written in English and published between 2015 and 2025. Studies that were excluded involved patients who underwent mastectomy without BCS, individuals without a history of breast cancer, patients without breast edema, male patients with breast cancer, or those younger than 18 years. In addition, opinion pieces, editorials, animal studies, and non-research articles were not considered. The review was therefore restricted to clinical and observational research conducted in human populations within healthcare settings.

Data Sources and Search Strategy

A comprehensive literature search was performed in September 2025 using three electronic databases: Ovid MEDLINE, EMBASE, and Web of Science. An initial, targeted search was conducted to identify articles on the topic. Text words from the titles and abstracts of relevant articles, along with index terms, were used to construct a comprehensive search strategy. Search strategies were developed using combinations of keywords and controlled vocabulary related to breast edema, breast swelling, breast lymphedema, and BCRL. Boolean operators were used to combine terms, including “breast edema,” “breast oedema,” “breast swelling,” and “breast lymphedema” with “lymphedema” or “BCRL.” In EMBASE, both Emtree subject headings and free-text terms were used to capture studies related to breast neoplasms, BCS, lumpectomy, radiotherapy, edema, and lymphedema. Searches included synonyms, and truncated terms were appropriate. The reference list of all included sources of evidence was screened for additional relevant articles. 

Study Selection

Study selection was performed using Rayyan, a web-based systematic review screening tool (Rayyan Systems Inc., Cambridge, MA, USA) [30]. Two reviewers (Reviewer A and Reviewer B) independently screened titles and abstracts (Tier I screening), with a third reviewer resolving any disagreements through discussion. A separate pair of reviewers (Reviewer C and Reviewer D) independently conducted a full-text review of potentially eligible articles (Tier II screening). Any disagreements regarding the inclusion or exclusion of full-text reviews were resolved through discussion between the two reviewers until consensus was reached.

Critical Appraisal

After completion of Tier II screening, all remaining articles were critically appraised using the Joanna Briggs Institute (JBI) critical appraisal checklists appropriate to each study’s methodology (e.g., cross-sectional, cohort, randomized controlled trial (RCT), etc.) [31]. A total of 26 articles were independently evaluated by two reviewers, with any discrepancies resolved through discussion to ensure consistency across all reviewed publications. Studies with unclear reporting, high risk of bias, ethical concerns, or major methodological limitations were excluded from the final review. This appraisal process ensured that only studies demonstrating adequate methodological rigor, relevance, and transparency were included in the final synthesis. Following critical appraisal, three articles were excluded due to a high risk of bias. Thus, 23 articles remained included in the final synthesis.

Data Extraction and Synthesis

Data extraction was performed independently by two reviewers using a standardized data extraction form in Google Docs. Through an iterative process, team members independently charted the data, discussed the results, and continuously updated the data extraction form. The information extracted focused on the study characteristics (author, year, setting/country), aim of study, sample size, methods, main findings, strengths, limitations, and conclusion of the final articles included in the review. Any disagreements about whether to include or exclude a full-text article were resolved among the two reviewers; no additional reviewer was needed. The final draft underwent editing and review by all the remaining authors. Any global discrepancies were addressed collectively among all authors through discussion and debate. Data were synthesized narratively and organized thematically. A summary table was used to compare study design, setting, population, limitations, key outcomes, and conclusions. Findings were reported with reference to the review question.

Results

Study Selection

**Figure 1:**
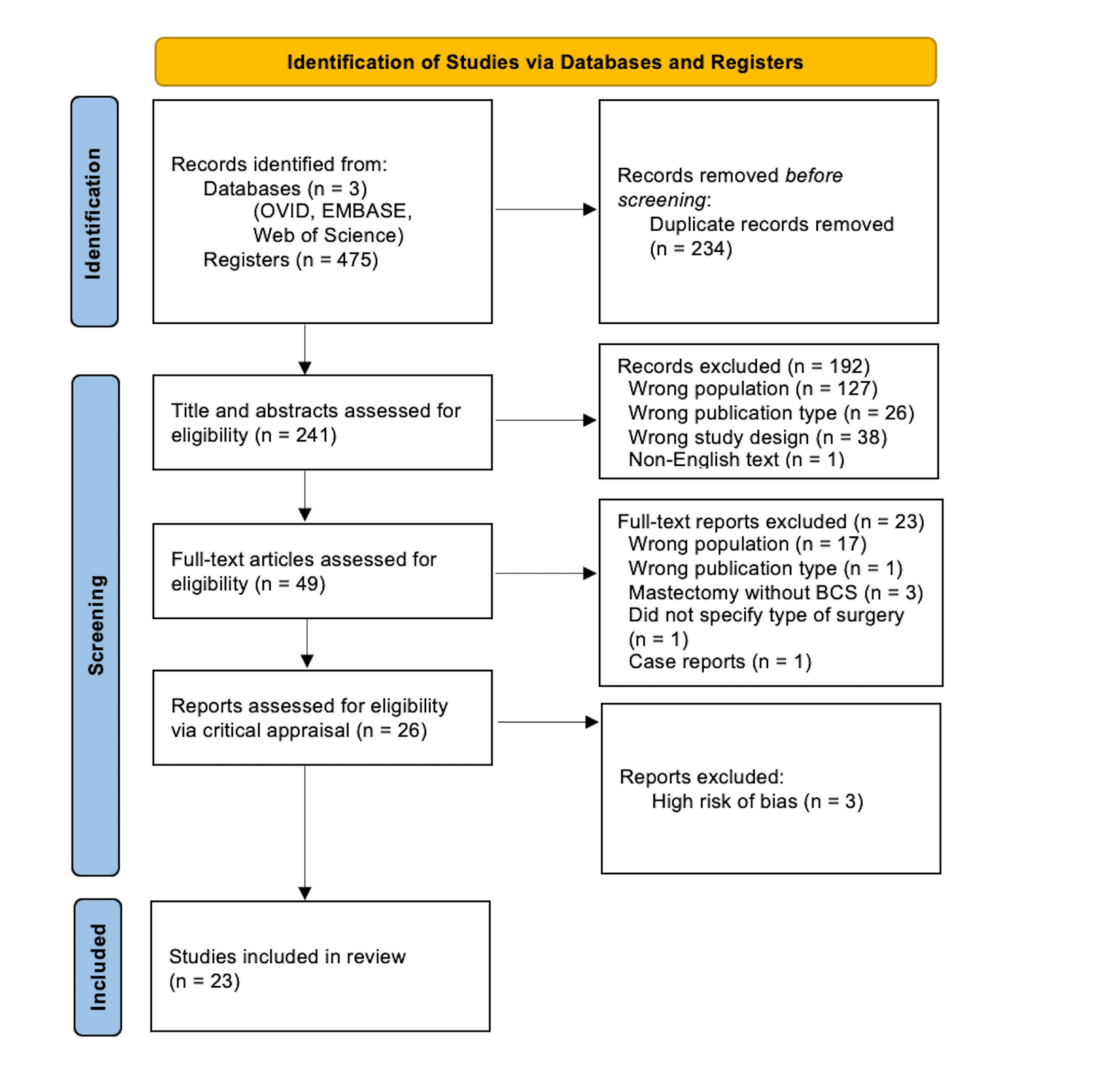
PRISMA flow diagram of study selection process. Preferred Reporting Items for Systematic Reviews and Meta-Analyses (PRISMA) 2020 flow diagram of study selection. Of 475 records identified through database searching, 234 duplicates were removed. After Tier I screening of 241 titles and abstracts, 49 full-text articles were assessed for eligibility. Following critical appraisal, three articles were excluded due to a high risk of bias, and 23 studies were included in the final synthesis. BCS: breast-conserving surgery

A total of 475 records were retrieved across the three databases. After removing 234 duplicates, 241 titles and abstracts were screened. Of these, 192 articles were excluded due to the following reasons: wrong population (n=127), wrong publication type (n=26), wrong study design (n=38), and non-English text (n=1). The remaining 49 full-text articles were assessed for eligibility. From the 49 articles, 23 articles were excluded for the following primary reasons: wrong population (n=17), wrong study design (n=1), mastectomy without BCS (n=3), did not specify the type of surgery (n=1), and case report (n=1). The remaining 26 articles were independently appraised using the JBI checklist by two reviewers. Following the appraisal, three articles were excluded due to a high risk of bias, leaving 23 articles that met the final inclusion criteria. Figure 1 presents the PRISMA flow diagram of the study selection process.

Study Characteristics

The final 23 included studies were published between 2015 and 2025. Most studies were conducted in the United States (n=6), Greece (n=1), Sweden (n=2), Israel (n=1), Italy (n=2), Turkey (n=1), Switzerland (n=1), China (n=1), Belgium (n=1), the Netherlands (n=1), Germany (n=3), Denmark (n=1), Japan (n=1), and Egypt (n=1). All included studies employed quantitative study designs. Sample sizes ranged from 11 to 1,640 participants, with most studies focusing on women who underwent BCS. These studies represented a combined sample of 10,402 participants across 14 countries. Most studies were cohort studies (n=15), with the remaining comprising observational studies (n=2), pilot studies (n=1), clinical trials (n=1), case series (n=1), cross-sectional studies (n=1), quasi-experimental designs (n=1), and RCTs (n=1). Using the JBI checklist, the methodological quality of the included studies was rated moderate to high. The characteristics of the included studies are summarized in Table 1.

**Table 1:** Summary of studies evaluating breast edema following breast-conserving surgery (BCS). The table summarizes study aims, design, sample characteristics, key findings, and limitations. ALNC: axillary lymph node clearance, ALND: axillary lymph node dissection, ANOVA: analysis of variance, BCCT.core: breast cancer conservative treatment cosmetic outcome software, BCS: breast-conserving surgery, BCRL-B: breast cancer-related lymphedema of the breast, BIBCQ: Body Image after Breast Cancer Questionnaire, BLE: breast lymphedema, BMI: body mass index, CTCAE: Common Terminology Criteria for Adverse Events, DASH: Disabilities of the Arm, Shoulder and Hand questionnaire, DCIS: ductal carcinoma in situ, EBRT: external beam radiotherapy, EORTC: European Organisation for Research and Treatment of Cancer, FAST: ultra-hypofractionated radiotherapy protocol, Gy: gray (unit of radiation dose), GWAS: genome-wide association study, HF-WBI: hypofractionated-whole-breast irradiation, HR-QoL: health-related quality of life, ICG: indocyanine green, ICG-A: indocyanine green angiography, IORT: intraoperative radiotherapy, IORT-b: intraoperative radiotherapy boost, IORT-f: full-dose intraoperative radiotherapy, LeQOLiS: Lymphedema Quality of Life Score, LENT-SOMA: Late Effects in Normal Tissues-Subjective, Objective, Management, Analytic scale, LTW: local tissue water, NCI: National Cancer Institute, ORM: oncoplastic reduction mammoplasty, QOL: quality of life, RT: radiotherapy, SeB: sequential boost, SIB: simultaneous integrated boost, SLNB: sentinel lymph node biopsy, SLND: sentinel lymph node dissection, SNP: single nucleotide polymorphism, START-B: Standardization of Breast Radiotherapy B trial protocol, TDC: tissue dielectric constant, TEWL: transepidermal water loss, TWEL: transepidermal water loss measurement, VAS: visual analog scale, WBI: whole-breast irradiation

Author, year	Study aim	Study setting and country	Sample description	Study design and methods	Study main findings	Study limitations and strengths	Study conclusions
Young-Afat et al., 2019 [5]	Evaluate patient-reported prevalence, risk factors, and effect of breast edema on health-related quality of life (HR-QoL) and breast pain in women undergoing BCS	Single center, University Medical Center (UMC) Utrecht, the Netherlands	836 patients undergoing BCS followed by RT.	Type: prospective cohort study. Patient-reported outcomes were collected via EORTC QLQC30/BR23 questionnaires at multiple time points (baseline at start of RT, three months then at 6, 12, and 18 months).	24.8% reported breast edema at some point and prevalence was the highest at six months (12.4%). Risk factors associated with breast edema were larger tumor size, oncoplastic surgery, ALND, locoregional RT, RT boost, and adjuvant chemotherapy HR-QoL: breast edema was associated with more breast pain, and poor QOL, physical functioning, and body image.	Limitations: patient-reported outcomes measured for breast edema (swelling of the breast) was not specific. Strengths: longitudinal data collection at multiple times over 18 months.	Breast edema is a frequent complication after BCS, and it peaks around six months post-RT. Breast edema is associated with several treatment factors (tumor size, oncoplastic surgery, ALND, locoregional RT, RT boost, and adjuvant chemotherapy) and has a significantly negative impact on patients’ QoL and breast pain.
Kedar et al., 2025 [8]	Type/extent of axillary surgical intervention (SLNB, lymph node sampling, or ALND) following BCS and its relationship to breast edema and arm lymphedema	Sourasky Medical Center Tel Aviv, Israel	105 female patients with breast cancer who underwent BCS with different axillary interventions.	Type: retrospective cohort study Breast edema diagnosed by a doctor during physical exam: visible swelling, tenderness, or peau d’orange Objective: measurement of arm lymphedema (key predictor) using arm circumference and volume.	Overall breast edema prevalence: 7.6%. No patient with SLNB developed breast edema vs. 23.5% of patients with ALND.	Limitations: small sample size and single-center design limited the generalizability.	Highlights that surgical extent influences the risk of breast edema and significant predictors include arm lymphedema, BMI, tumor grade 50% of cases: co-occurrence of breast and arm lymphedema observed.
Cornacchia et al., 2022 [32]	Describe the incidence of breast cancer-related lymphedema of the breast (BCRL-B) after BCS and identify the risk factors for development of postoperative breast edema.	Single-center (IRCSS Ospedale Policlinico San Martino), Genoa, Italy	530 female patients with early-stage breast cancer who underwent BCS. Subgroup of patients who developed BCRLB (n=31).	Type: retrospective cohort study during COVID-19. A questionnaire via telephone was used to screen BCRL-B symptoms. Thirty-one patients underwent objective measurement of breast edema using tissue dielectric constant (TDC) to calculate local tissue water (LTW) ratio and assess skin induration/fibrosis using SkinFibroMeter.	Incidence of BCRL-B was 5.8%. Patients who underwent lumpectomy + axillary lymph node dissection had higher chances of developing breast edema compared to those who had lumpectomy + sentinel node biopsy (29% vs. 12%). Second axillary surgery to remove more nodes also significantly increased the risk, 25.8% of patients with breast edema showed signs of tissue fibrosis.	Limitations: retrospective identification of patients with BCRL-B via a telephonic questionnaire may have introduced selection bias; no control group. Strengths: included both patient-reported symptoms and objective, quantitative measures to diagnose breast edema.	Early diagnosis with tools such as TDC measurement may be crucial for improving the patients’ QoL. Primary risk factor for BCRL-B is the extent of axillary surgery.
Yono et al., 2025 [33]	Define the incidence of breast lymphedema (BLE) and identify factors associated with the risk of developing chronic BLE in women undergoing BCS.	Single Quaternary Care Cancer Institute, Detroit, Michigan, United States	1,052 female patients with a new diagnosis of early-stage breast cancer who underwent BCS followed by adjuvant whole-breast radiation therapy.	Type: retrospective cohort study Patients were divided into two groups: those who developed BLE (n=99) and those who did not (n=953). The incidence of BLE was defined as visible breast swelling>one year after RT completion. Univariate and multivariate regression analyses were used to identify risk factors: patient demographics, clinical characteristics (breast volume, BMI, and race), treatment factors (chemotherapy, axillary surgery, and radiation dose), postoperative complications (cellulitis, seroma, and arm lymphedema).	The incidence of BLE was 9.4% (99 patients out of 1,052). Significant risk factors for BLE: preoperative breast volume>1500 cm³, Black race, neoadjuvant chemotherapy, adjuvant chemotherapy, postoperative cellulitis, and pre-existing arm lymphedema.	Strengths: large sample size and a long follow-up of 51 months, diverse patient population, and quantitative measurement of breast volume. Limitations: retrospective and single-center setting.	Patients with larger breast volumes>1500 cm³, Black patients, given chemotherapy, and those who developed arm lymphedema or cellulitis were at a significantly higher risk for BLE after BCS. High-risk patients require increased monitoring to prevent BLE. Required further investigation into the racial disparity in BLE risk.
Unterkirhere et al., 2023 [34]	Assess the treatment outcomes of patients who received a condensed three-week radiation treatment that delivered a boost dose to the tumor area and whole breast at the same time.	Single-center (Kantonsspital Luzern Lucerne), Switzerland	424 female patients with stage I-III invasive or ductal carcinoma in situ who underwent BCS.	Type: prospective cohort study Treatment toxicity (acute and late), patient-reported outcome measures, disease control incidence, and grade of breast edema and arm lymphedema.	Breast edema: medical treatment for breast edema (grade 3 late effect) was required for 11 patients. Patients who developed breast edema had a significantly larger median breast volume.	Limitations: reports on breast edema as a secondary outcome in a broad treatment cohort.	Useful data on low-incidence breast edema requiring medical intervention following this specific hypofractionated RT protocol.
Ganju et al., 2019 [35]	Assess the incidence of breast lymphedema (BLE) in women with early-stage breast cancer treated with lumpectomy (BCS) and whole-breast RT and identify predictors for its development.	Single-center, University of Kansas School of Medicine/University of Texas (MD Anderson Cancer Center), United States	230 female patients with early-stage breast cancer who underwent BCS and adjuvant whole-breast RT	Type: retrospective cohort study Data on patient demographics, tumor characteristics, and treatment were extracted from medical records. BLE was physically diagnosed and graded (mild, moderate, and severe) by nurses in a dedicated lymphedema clinic. Identification of associations using univariate analysis (chi-square tests).	18.7% developed BLE, 93% of cases were grade 1 (mild), 7% were grade 2 (moderate), and no severe cases. 61% resolved by last follow-up with conservative treatment (compression and manual lymphatic drainage). HER2 + tumor subtype was significantly associated with BLE development, and a trend was observed for trastuzumab use. No significant associations found between BLE and age, T-stage, number of nodes removed, arm lymphedema, or chemotherapy.	Limitations: retrospective single institution design, lack of objective quantitative measures for the diagnosis of BLE. Strengths: standardized assessment in a dedicated lymphedema clinic.	Conservative treatment for BLE is often effective. Tumor biology, such as HER2+ subtype, may be a more significant factor of BLE development than surgery or RT.
Rivera et al., 2025 [36]	Compare the efficacy (survival and relapse) and safety of ultrafractionated (FAST protocol) between moderate hypofractionated (START-B protocol) locoregional RT in elderly patients with node-positive breast cancer.	Single radiotherapy center, (CHU UCL Namur), Belgium	221 female patients aged > 65 years with non-metastatic, node-positive breast cancer. FAST group: n=89 (40 underwent BCS) START-B group: n=132 (75 patients underwent BCS).	Type: retrospective cohort study, comparative. Clinical FAST: 28.5 Gy in five fractions, once per week for five weeks START-B: 40 Gy in 15 fractions, five days per week for three weeks. Data were extracted from medical records.	No significant differences in overall survival or disease survival (efficacy). No significant differences in acute dermatitis or arm edema. Significantly higher risk of moderate breast edema in the FAST group.	Limitations: retrospective design (bias potential), limited sample size.	FAST protocol for locoregional irradiation in elderly patients with node-positive breast cancer showed no significant difference in the efficacy compared to the START-B protocol. FAST protocol: higher risk of breast edema.
Koukourakis et al., 2021 [37]	Report long-term results (12 year follow-up) of a hypofractionated accelerated radiotherapy after BCS (safety, efficacy, toxicity, and oncologic outcomes).	Single-center (University Hospital of Alexandroupolis), Greece	367 female patients with breast cancer treated with BCS.	Type: prospective single-center clinical trial (non-randomized) two HypoAR schemes with a tumor bed boost: amifostine was offered to 252 patients. Early assessed with NCI CTCAE v5 (dermatitis, breast edema, pain, and pneumonitis). Late assessed with modified LENTSOMA (breast edema, fibrosis, telangiectasia, arm lymphedema, and lung fibrosis).	Minimal early and late toxicity (symptomatic breast edema (2.2%) and fibrosis (1.9%)). Low incidence of carcinogenesis. Amifostine (chemotherapy drug) significantly reduced acute radiation dermatitis.	Strengths: long-term follow-up (median-12 years), detailed toxicity analysis. Limitations: nonrandomized, single-center, scheme selection by physician discretion.	Supports safety/efficacy of HypoAR with boost=very low rates of breast edema and other late toxicities over long-term. HypoAR is a safe and effective postopertaive treatment for breast cancer with a low risk of carcinogenesis.
Kerrigan et al., 2022 [38]	Assess the utility of ultrasound-measured difference in the dermal thickness between the affected and unaffected breast as an objective measure of breast lymphedema. Associate the measure with patients’ characteristics, treatments, and patient-reported QoL.	University of Vermont Medical Center, Vermont, United States	40 patients with breast cancer. Intervention group (n=30): females with unilateral invasive breast cancer treated with BCS, SLNB, and RT. Control group (n=10): females with benign breast disease with no previous breast surgery/RT.	Type: prospective pilot study Dermal thickness difference was measured using bilateral breast ultrasound, and breast lymphedema was defined as a difference of>0.3 mm between the affected and unaffected breast. Associations between patient and treatment factors such as breast lymphedema, tumor size, number of lymph nodes removed, breast density, and BMI were assessed. Impact on QoL measured using the modified DASH questionnaire.	63% of invasive group had breast lymphedema (dermal thickness difference>0.3 mm). Breast lymphedema was associated with larger tumor size, more lymph nodes removed, larger breast volume irradiated, and receipt of radiation boost. Also, significantly correlated with worse sleep, confidence, social life, and work function. Ultrasound measurements correlated with clinical exam findings.	Limitations: small sample size in a single center and lack of racial diversity (White, rural population), and no baseline dermal thickness data. Strengths: included a control group to establish the objective, diagnostic threshold; correlated objective measures with physical exam findings and patient-reported outcomes.	Provides a foundation for an objective breast lymphedema definition. Ultrasound was used to measure the difference in the dermal thickness; it is accessible, inexpensive, and an objective method to quantity breast edema.
Chen et al., 2024 [39]	To explore and develop classification systems for breast lymphedema (BLE) using breast ultrasound, physical exam findings, and patient-reported outcomes in a racially diverse cohort.	Two academic medical centers in the USA: University of Vermont Medical Center (Burlington, VT) and Rush University Medical Center (Chicago, IL)	80 women: 60 with a history of unilateral invasive breast cancer treated with breast- conserving surgery (BCS), sentinel lymph node biopsy (SLNB), and radiotherapy (at risk for BLE), and 20 control patients with benign breast complaints. The cohort was racially diverse, with 23 participants from communities of color.	Type: cross-sectional study Data were collected by point-of-care ultrasound (measuring dermal thickness), physical exam, and a modified patient-reported outcome questionnaire (DASH). Two classification approaches were used: 1) a simple ultrasound-based metric (dermal thickness difference≥0.5 mm) and 2) an unsupervised machine learning algorithm (Kohonen self-organizing map-SOM) to create a multi-parameter classifier.	The prevalence of ultrasound-defined BLE (dermal thickness difference ≥0.5 mm) among patients with invasive breast cancer was 72%. BLE prevalence was lower in women from communities of color (61%) than in White women (78%); however, the distribution of dermal thickness differences suggested potentially more severe BLE in women of color. The SOM classifier identified three distinct patient clusters: Cluster 1 (severe BLE, 15% of patients), Cluster 2 (moderate BLE), and Cluster 3 (little/no BLE). White patients were more likely to report a negative impact on QOL despite the potential for less severe objective BLE measurements compared to women of color.	Limitations: small sample size, potential geographic confounding (sites had different racial distributions), use of different ultrasound instruments, use of a non-validated, modified QOL questionnaire. Strengths: racially diverse cohort, use of an objective, accessible measurement tool (ultrasound), novel multi-parameter classification approach combining objective and subjective measures.	Ultrasound-based dermal thickness measurement is an objective and transportable method for quantifying BLE. A multi-parameter classifier combining ultrasound, physical exam, and patient-reported outcomes shows promise for a more nuanced classification of BLE. Racial disparities exist in both the prevalence and potential severity of BLE, as well as in the reporting of its impact on QOL. Prospective studies in larger cohorts are needed to validate these classifiers.
Ibrahim et al., 2023 [40]	To identify the risk factors for breast lymphedema (BLE) and assess the utility of breast ultrasound for diagnosing BLE	Fayoum University Hospital, Egypt	286 female patients with breast cancer treated with wide local excision. Mean age: 54.7 years, mean BMI: 31.5 kg/ m^2^, mean breast volume: 1223 mL. All received adjuvant whole- breast radiotherapy.	Type: retrospective case series Design: Patients were assessed clinically and with ultrasound at 6 and 12 months postsurgery. BLE was defined as clinical skin edema plus>5 mm added skin/subcutaneous thickness on ultrasound compared to the contralateral breast.	BL incidence was 7.7% (22/286 patients). Significant risk factors included higher BMI, larger breast volume, upper outer quadrant tumors, and axillary lymph node clearance (ALNC). Ultrasound showed persistent increased skin/subcutaneous thickness in BLE patients at 6 and 12 months. No significant association was found with age, tumor side, or biology.	Strengths: relatively large sample size, objective ultrasound criteria for diagnosis. Limitations: single-center, retrospective; no pre-radiotherapy ultrasound; breast density not assessed; limited follow-up.	Breast lymphoedema is a common complication with an incidence of 7.7% in this cohort. Ultrasound can aid the diagnosis by detecting >5 mm added skin/subcutaneous thickness. High BMI, larger breast volume, upper outer quadrant tumors, and ALNC are significant risk factors.
Lauritzen et al., 2023 [41]	To investigate the feasibility of using indocyanine green angiography (ICG-A) in oncoplastic breast-conserving surgery (OBCS) to locate perforators and assess tissue perfusion, and to correlate intraoperative ICG-A findings with postoperative complications (infection, skin necrosis), scar quality, quality of life, timing of adjuvant therapy, and lymphedema	Copenhagen University Hospital and Herlev Gentofte Hospital, Denmark	11 patients with breast cancer (from 15 initially included). Mean age: 59.2 years (range: 47-71). Mean BMI: 25.9 kg/m² Surgical types: 7 volume displacement, 4 volume replacement procedures (including LICAP and msLD flaps).	Type: prospective observational study Design: ICG-A was used at three intraoperative timepoints (after lumpectomy, after perforator dissection, and after wound closure). Patients were followed clinically at four weeks, four to six months, and 12 months. Assessments: ICG-A perfusion, BREASTQ, POSAS, lymphedema measurements, complication rates.	ICG-A successfully located perforators and confirmed sufficient perfusion in 100% of cases. No postoperative necrosis or major complications occurred. One patient (9%) had minor complications (infection/seroma). 36.4% (4/11) developed breast edema (all on the irradiated side). No arm lymphedema was observed. Patient-reported scar satisfaction was lower than surgeon assessment at four weeks and four to six months but equalized by 12 months. The quality of life improved significantly during follow-up. Adjuvant therapy was timely in 91% of patients.	Strengths: Prospective design, standardized ICGA protocol, comprehensive follow-up, use of validated patient-reported outcome measures. Limitations: Small sample size, single-center study, no control group, limited generalizability.	ICG-A is feasible and effective for intraoperative perfusion assessment in OBCS, aiding in perforator selection and flap design. It corresponded with clinical evaluation and was associated with no postoperative necrosis. Breast edema was common in irradiated breasts. Larger studies are needed to explore ICG-A’s impact on complications and outcomes in high-risk OBCS cases.
Hannoudi et al., 2025 [42]	To assess whether women with macromastia who received oncoplastic reduction mammoplasty (ORM) have a reduced incidence of postoperative breast lymphedema compared to those who received breast- conserving surgery (BCS) alone.	Single large metropolitan cancer institute in Southeast Michigan, USA	782 women who underwent BCS alone (n=718) or ORM (n=64) followed by whole-breast radiation between 2016 and 2023. Macromastia was defined as preoperative breast volume ≥1500 cm³.	Type: retrospective cohort study. Data were collected via chart review. Statistical analyses included univariate and multivariate regression to compare outcomes and identify factors associated with breast lymphedema.	Overall breast lymphedema incidence: 10.6%. Patients with breast volume≥1500 cm³ who underwent BCS alone were 6.575 times more likely to develop breast lymphedema than those who underwent ORM (p=0.014). Black race, ALND, cellulitis, and arm lymphedema were also positively associated with breast lymphedema. ORM was associated with larger negative margins and lower re-excision rates but higher wound complications and hematoma rates.	Limitations: small ORM subgroup (n=64), potential detection bias due to shorter follow-up in the ORM group, and estimated breast volume measurements. Strengths: large overall sample size, use of multivariate analysis to control for confounders, and novel focus on ORM’s role in preventing breast lymphedema.	ORM may reduce the risk of breast lymphedema in women with macromastia (breast volume ≥1500 cm³) compared to BCS alone. It should be considered as a preventive surgical option for eligible patients.
Sorrentino et al., 2018 [43]	Compare local recurrences, toxicities, self-assessment of body image, and return to work or daily activities between three RT approaches: External beam RT (EBRT), full-dose intraoperative RT (IORT-f), and IORT boost (IORT-b).	Single-center, (ICS Maugeri Hospital), Pavia, Italy	443 female patients who underwent BCS. EBRT group: 220 patients. IORT-f group: 140 patients. IORT-b group: 83 patients.	Type: prospective nonrandomized cohort study. Patients were divided into treatment groups based on specific clinical criteria. Data were collected on toxicities, patient-reported outcomes via Body Image after Breast Cancer Questionnaire (BIBCQ) and a custom questionnaire on work/daily activities.	EBRT group had higher overall risk of complications and specific risks of fibrosis, breast edema, and pain compared to the IORT-f group. The IORT-b group had a higher risk of seroma BIBCQ scores: IORT-f patients reported significantly better outcomes in arm concerns. IORT-f allowed the fastest return to work/activities compared to the other two groups.	Limitations: nonrandomized (selection bias between groups), short follow-up of 26-37 months, single-center study, questionnaire on work/daily activities was not validated. Strengths: comprehensive comparison of three modern RT techniques, inclusion of patient-reported outcomes and socio-economic factors.	Use of IORT-f treatment showed reduction in specific adverse effects related to whole-breast irradiation and also allowed significantly faster return to work and daily activities. EBRT patients had a significantly higher risk of breast edema and pain than IORT-f patients.
Wennman-Larsen et al., 2015 [44]	Examine the severity and development of breast and arm symptoms separately during first two years after BCS. Identify associated predictors of arm symptoms and breast symptoms	Three hospitals, Stockholm, Sweden	645 female patients who underwent BCS (67%) and SLND (56%) with 82% scheduled for postoperative RT.	Type: prospective cohort study Patients completed validated questionnaires (EORTC QLC-BR23) at baseline (within 12 weeks of BCS) and at 4, 8, 12, 18 and 24 months.	Breast symptoms were more severe than arm symptoms at points in time. Predictors of breast symptoms at two years: breast symptoms at eight months was the strongest predictor and a BMI of >25 kg/m^2^. Predictors of arm symptoms at two years: RT, arm symptoms at baseline and eight months and a BMI of >25 kg/m^2^.	Limitations: use of only self-reported measures for symptoms; dropouts during the follow-up period (selection bias). Strengths: three hospitals, prospective design with six measurement points over a two-year follow-up; data collection started soon after surgery.	Breast and arm symptoms should be considered separately after surgery. Early symptoms at eight months and a BMI >25 kg/m^2^ are strong risk factors for long-term symptoms in both breast and arm breast symptoms (pain, swelling, oversensitivity, and skin issues) were more severe than arm symptoms at all six points of time.
Hille-Betz et al., 2016 [45]	To identify risk-modifying factors for late radiation side effects, cosmetic outcomes, and pain in patients with breast cancer after breast-conserving surgery and 3D conformal radiotherapy.	Hannover Medical School, Germany	159 female patients with breast cancer (stage I-III or DCIS). Median age: 58 years (range 36-86). All were treated by a single breast surgeon. Surgery types: segmentectomy (n=85) or oncoplastic surgery (n=74). Received 3D-CRT between 2006 and 2013.	Type: retrospective cohort study Design: analysis of late toxicity (LENTSOMA), cosmetic outcomes (BCCT.core software), and pain (EORTC QLQ-BR23) Statistical analysis: multivariate logistic regression to identify significant predictors.	Arm lymphoedema (8.8%): significantly associated with axillary clearance (OR: 4.37, p=0.011). Breast edema (5.0%): associated with axillary clearance (OR: 10.59, p=0.004) and large/ptotic breasts (OR: 5.34, p=0.029). Poor cosmetic outcome (14.4%): associated with large/ptotic breasts (OR: 3.19, p=0.019). Breast pain (15.1%): trend toward association with total radiation dose including boost (OR: 1.077, p=0.060). Arm/shoulder pain (21.4%): significantly associated with arm lymphoedema (OR: 3.9, p=0.027). No significant association between radiotherapy parameters and late toxicities (except breast pain trend).	Strengths: homogeneous patient group (single surgeon), use of objective cosmetic assessment software (BCCT.core) Limitations: retrospective design, single follow-up assessment, no pre-radiotherapy cosmetic evaluation, potential selection bias	Axillary clearance is a key risk factor for arm and breast lymphoedema. Large and ptotic breasts are associated with poor cosmetic outcomes and breast edema. Radiotherapy parameters (except the total dose for breast pain) did not significantly influence late side effects. Modern 3D-CRT techniques may reduce radiotherapy-related morbidity.
Wang et al., 2025 [46]	Compare safety and efficacy of two RT boost techniques: simultaneous integrated boost (SIB) and sequential boost (SeB) during hypofractionated whole-breast irradiation (HF-WBI) after BCS	Single-center, National Cancer Center, Beijing, China	1,132 female patients with stage pT1-3 N0-3 M0 breast cancer who underwent BCS. SIB group: 775 patients. SeB group: 357 patients.	Type: prospective randomized comparative study (two trials). Patients received HF-WBI; tumor bed received either SIB (43.5 Gy in 15 fractions) or SeB (8.7 Gy in three fractions). Outcomes compared between two groups.	No significant differences were found in five-year local control, disease-free survival, or overall survival between SIB and SeB groups. No significant differences in clinically relevant toxicities, including breast swelling, skin toxicity, pain, lymphedema, or pneumonitis.	Limitations: nonrandomized Strengths: large sample size.	SIB is an effective alternative SeB because it offers comparable oncologic outcomes, toxicity profiles, and cosmetic results while providing the advantage of a shorter overall treatment duration (three fractions compared to 15). No significant difference was found in the rates of breast swelling between both groups.
Goerdt et al., 2024 [47]	To evaluate acute and long-term toxicity profiles in patients with high-risk breast cancer receiving an intraoperative radiotherapy (IORT) boost with low-energy X-rays followed by whole-breast irradiation (WBI).	Multicenter study across 10 German centers	1,133 registered patients (10 of them had two carcinomas, resulting in a total of 1,143 carcinomas). Of these, 902 patients, corresponding to 910 carcinomas, were included in the final analysis. Median age: 61 years (range: 30-90). All had tumors≤3.5 cm and preoperative indication for boost radiotherapy.	Type: prospective multi-center registry study (phase IV) Design: patients received IORT boost (20 Gy) during breast-conserving surgery, followed by EBRT (median: 50.4 Gy). Toxicity assessment: LENT-SOMA criteria at predefined intervals up to 10 years. Analysis: intention-to-treat, cumulative toxicity rates calculated using the Kaplan-Meier method.	Acute toxicity (≤90 days): no grade 3-4 toxicities; grade 1-2 erythema (4.4%), palpable seroma (9.1%), wound healing disorders (2.1%). Chronic toxicity (cumulative).	Strengths: large prospective multi-center cohort, long follow-up (up to 10 years), detailed toxicity documentation, real-world registry setting. Limitations: limited to German population, mostly normofractionated EBRT (not current hypofractionation standard), attrition over time (20.4% dropout), investigator-initiated trial without external funding.	IORT boost with low kV X-rays is safe and feasible, with low rates of acute and chronic toxicity. It represents a high quality standard for boost delivery in patients with high-risk breast cancer undergoing breast-conserving therapy.
Tuğral et al., 2023 [48]	Measure acute changes in skin properties (local tissue water (edema) and skin barrier function) before and after RT as potential early indicators of breast lymphedema risk	Single center, Izmir, Turkey	24 female patients with breast cancer who were candidates for adjuvant radiotherapy after surgery (BCS or axillary lymph node dissection).	Type: prospective observational study Patients were evaluated before and after completing RT. Skin properties were measured at four points (two contralateral + two ipsilateral) using TDC to quantify local tissue water and TWEL to assess skin barrier function.	Significant increase in tissue water (TDC) was found in the upper breast at the 5.0 mm depth after RT. Skin barrier function (TEWL) was significantly worsened in the axillary region after RT but not at specific points in the breast. Significant correlations found between EWL and TDC values at the axillary site after RT.	Limitation: single setting and small sample size with lack of diversity, short-term effects of RT in breast lymphedema development. Strengths: use of objective, non-invasive tools (TDC and TEWL) to quantify biophysical changes and detailed evaluation at multiple sites and tissue depths.	Study’s purpose is that exposure to early biomarkers of risk of breast lymphedema RT led to a significant deterioration in skin barrier function in the axilla region and increased local tissue in upper breast at the deep tissue level. Early detection of RT-induced changes can be assessed with non-invasive devices such as TDC and TEWL.
Johansson et al., 2020 [49]	Examine if compression therapy (using sports bra) was effective as an early treatment to reduce breast edema symptoms compared to standard bra	Single center, Department of Oncology, Skane University Hospital, Lund, Sweden	56 female patients with breast cancer treated with BCS and RT; all with breast edema diagnosed post-RT (TDC ratio >1.40). Intervention group: (n=28). Control group: (n=28).	Type: randomized controlled pilot study (two-arm parallel group) Intervention group: wore compressive sports bra during daytime for nine months. Control group: wore standard bra. Tissue water content was measured by TDC and patient outcomes assessed using the Visual Analoge Scale (VAS).	No significant differences were found between intervention and control groups for TDC values or VAS scores. Both groups: there was significant reduction in breast tissue water content (TDC values) and experience of heaviness (VAS) and proportion of patients experiencing tightness and heaviness, and pain decreased over time.	Limitations: pilot study with small sample size and intervention, was not blinded to participants. Strengths: used an objective tool (TDC) to measure breast edema.	The use of compression treatment with a sports bra showed no significant difference over standard bra in reducing breast edema or symptoms. Suggests natural resolution over time.
Pez et al., 2020 [50]	To evaluate long-term oncological outcomes and chronic side effects in patients with breast cancer receiving intraoperative radiotherapy (IORT) as an upfront boost	Single-center study at University Medical Center Mannheim, Germany	400 consecutive patients with breast cancer. Median age: 63 years (range: 30-85). Tumor diameter ≤3.5 cm. Included high- risk features: 26.5% T2 tumors, 24.3% node-positive, 22% grade 3.	Type: retrospective analysis with prospective toxicity assessment Design: patients received IORT boost (20 Gy using low-energy X-rays) during breast-conserving surgery, followed by whole-breast irradiation (46-50 Gy). Follow-up: median 78 months (range: 2-180 months). Toxicity assessment: LENT-SOMA scales; chronic toxicity defined as grade ≥2 occurring ≥three times during follow-up. Statistical analysis: Kaplan-Meier method for survival and toxicity rates.	Oncological outcomes: local recurrence rates: 2.0% (five years), 6.6% (10 years), 10.1% (15 years). Overall survival: 92.1% (five years), 81.8% (10 years), 80.7% (15 years). Chronic toxicity (≥grade 2): Fibrosis: 19.1% (five years), 21.2% (12 years). Pain: 8.6% (≥four years), breast edema: 2.4% (≥two years). Hyperpigmentation: 0.5% (≥two years). No chronic lymphedema observed. Most side effects occurred within the first three years.	Strengths: large sample size, long follow-up (up to 15 years), prospective toxicity documentation, detailed chronic toxicity analysis. Limitations: retrospective design, single-center experience, potential selection bias, no direct comparison group.	IORT boost with low-energy X-rays is an efficient and safe method for delivering a tumor bed boost in patients with high-risk breast cancer, providing excellent local control with acceptable long-term toxicity profiles comparable to or better than external beam boost techniques.
Jandu et al., 2023 [51]	To identify common single nucleotide polymorphisms (SNPs) associated with toxicity two years after whole- breast radiotherapy using a genome-wide association study (GWAS), and to validate previously reported SNPs	Multicenter, 18 radiation oncology centers across Europe and the United States	1,640 patients with breast cancer of European ancestry with complete genetic, clinical, treatment, and toxicity data. All had undergone breast-conserving surgery and adjuvant external beam radiotherapy.	Type: prospective cohort study (REQUITE consortium) Design: Genome-wide association study (GWAS) Data collection: toxicity assessed using CTCAE v4.0 at baseline, end of radiotherapy, and annually. Genotyping: Illumina OncoArray with imputation using the 1,000 Genomes Project reference. Analysis: multivariable logistic regression adjusted for clinical/treatment factors and population structure; genome-wide significance threshold: p<5×10-8.	Eight SNPs reached genome-wide significance for specific toxicity endpoints: nipple retraction, breast edema, induration, arm lymphedema. Heritability estimates ranged from 25% to 39% for significant endpoints. No replication of previously reported SNPs at the pre-specified significance level. Polygenic risk scores (PRS) were developed and associated with several toxicity endpoints.	Strengths: large, prospective, multicenter design; standardized toxicity assessment; rigorous genetic quality control. Limitations: modest sample size for a GWAS; limited power for rare variants and some endpoints; lack of diversity (European ancestry only); no replication of previous SNPs.	This GWAS provides evidence that common genetic variants are associated with specific late radiation toxicity endpoints in patients with breast cancer. The findings support the polygenic nature of radiation toxicity and highlight the potential for genetic risk prediction models to personalize radiotherapy in the future.
Yamamoto et al., 2022 [52]	To develop and evaluate a novel pathophysiological severity staging system for breast lymphedema (BLE) based on indocyanine green (ICG) lymphography findings and to assess its relationship with patient symptoms and quality of life (QOL)	Single-center; National Center for Global Health and Medicine, Tokyo, Japan	37 breast cancer survivors (all female, unilateral cancer) with subjective breast symptoms. Average age: 50.7 years. All had undergone breast cancer treatments (mastectomy or partial mastectomy, with axillary node procedures, chemotherapy, or radiotherapy).	Type: retrospective observational study Design: patients underwent breast ICG lymphography. A novel six-stage system (stages 0-V) was developed based on the visibility of linear lymphatic patterns and the extent of dermal backflow patterns (splash, stardust, and diffuse). Data collection: symptoms and lymphedema. Quality of Life Scores (LeQOLiS) were recorded and compared across ICG stages using chi-square tests and ANOVA.	Higher ICG stages were significantly associated with more frequent breast swelling (p=0.020) and cellulitis (p=0.024). Higher (worse) LeQOLiS (p<0.001).	Strengths: first study to propose a pathophysiological staging system for BLE; clear visualization of lymph flow; significant correlation with QOL. Limitations: small sample size (n=37); single ethnic group (Japanese); retrospective design; no assessment of long-term outcomes or intervention efficacy.	ICG lymphography effectively visualizes superficial breast lymph circulation. The proposed ICG staging system correlates with the prevalence of key BLE symptoms and patient QOL, providing a valuable tool for the diagnosis and severity assessment of breast lymphedema.

Prevalence of Breast Edema in Women With BCS

The reported prevalence of breast edema after BCS varied widely across studies, reflecting differences in outcome definitions, assessment methods, and follow-up duration. Across multiple clinical cohorts, the prevalence ranged from 5.8% to 24.8% during the first 12-18 months after treatment [5,32-36], with longitudinal assessment demonstrating the highest prevalence in the early posttreatment period followed by a decline over time [5]. The lowest reported prevalence, 2.7%, was observed in a study focusing on moderate acute breast edema [37]. Markedly higher prevalence was reported in US-based studies, including 63% with a dermal thickness difference >0.3 mm [38] and 72% with a ≥0.5 mm threshold [39]. In this study, the prevalence was lower in communities of color (40-79%) than in White patients (62-89%) [39]. A separate US cohort identified 7.7% patients with breast lymphedema and persistent breast thickness differences during the six-month and 12-month follow-ups [40]. In a small cohort of 11 patients, 36.4% developed breast edema within 12 months postoperative radiotherapy, with one of them developing it at the four to six months mark [41].

Procedure-specific prevalence was also noted, with breast edema occurring in 6.9% of patients who underwent lymph node sampling and in 23.5% who underwent axillary lymph node dissection (ALND), whereas none were observed among those who underwent sentinel lymph node biopsy (SLNB), giving an overall prevalence of 7.6% [8]. Similarly, among women with preoperative breast volumes ≥1500 cm^3^, 16.8% developed breast lymphedema after BCS compared to 4.1% who underwent oncoplastic reduction mammoplasty [42]. Only one study reported concomitant breast and arm lymphedema, in which 18.7% were diagnosed with breast lymphedema, and only three patients had both breast and arm lymphedema [32].

Comparison of Women With and Without Breast Edema

Across studies that directly compared women with and without breast edema, several clinical, surgical, and patient-reported differences were identified. In a large cohort, women with breast lymphedema were more likely to be Black, to have undergone ALND, and to have experienced postoperative complications, including cellulitis, hematoma, and seroma. Breast cancer-related arm lymphedema was also more common in this group (20.2% vs. 4.7%, p<0.001). However, after multivariate adjustment, only Black race, postoperative cellulitis, and chronic arm lymphedema remained independently associated with breast lymphedema [33]. Women with breast edema also had larger tumors than women without breast edema and experienced worse patient-reported outcomes, including poorer body image, lower quality of life, reduced physical functioning, and higher breast pain at all measured time points. These associations remained statistically significant after adjustment for confounding variables [5]. No significant differences were observed for age, BMI, number of resected lymph nodes, tumor characteristics, or receipt of systemic therapy and radiotherapy, although lumpectomy combined with ALND was more common among women who developed breast edema (29% vs. 12%) [32]. Assessments using US demonstrated that women with breast lymphedema had greater dermal thickness differences, larger affected-to-contralateral breast size ratios, and were more likely to exhibit abnormal clinical breast findings, such as pitting edema, nipple fullness, and changes in nipple texture [38]. Some studies reported breast-related symptoms or general treatment toxicity, but did not compare women with and without breast edema [43,44].

Treatment-Related Factors Associated With Breast Edema

The most consistently reported treatment-related factor was the extent of axillary surgery, with higher rates of breast edema observed following ALND compared to SLNB [5,8,32] and after axillary node clearance [40,45]. There was variability in the associations linked to radiotherapy. Locoregional radiotherapy was associated with breast edema at 3, 6, and 12 months, and radiotherapy boost was associated with breast edema at 18 months [5]. Larger irradiated breast volume and receipt of a radiation boost were associated with increased dermal thickness difference [38]. Use of an ultra-hypofractionated radiotherapy protocol was associated with a higher likelihood of moderate breast edema compared to the standard hypofractionated schedule [36]. Nodal irradiation was associated with a higher risk of any-grade breast edema, except for grade 2 breast edema [34]. In contrast, no differences were observed when comparing integrated and sequential boost techniques [46], and no associations were found with hypofractionated radiotherapy or radiation boost [37,32]. However, administration of amifostine was significantly associated with a reduction in acute breast edema (p=0.03) [37]. Neoadjuvant and adjuvant chemotherapy were independently associated with increased odds of developing breast lymphedema [33]. Specifically, adjuvant chemotherapy was associated with breast edema at 6, 12, and 18 months [5]. However, none of the patients in a small subgroup who received adjuvant radiotherapy developed breast edema [8].

Clinical and Patient-Related Risk Factors for Breast Edema

Multiple studies consistently associate breast edema with markers of lymphatic vulnerability and breast size. Larger preoperative breast volumes, higher BMI, ALND, and presence of BCRL were repeatedly identified as risk factors [8,33,34,40,42,45]. Postoperative cellulitis and tumor grade were also independently associated with breast lymphedema [8,33,42]. Sociodemographic and tumor-related factors showed more variable associations. The Black race was independently associated with higher odds of breast lymphedema in two large cohorts [33,42]. Tumor grade [8], tumor subtype [32], and tumor location [40] were each associated with breast lymphedema, whereas tumor T stage was not [32]. Age was consistently not associated with breast lymphedema [32,38,40,47]. Imaging-based studies further suggested weak associations between breast lymphedema and breast density, larger tumors, and the number of lymph nodes removed [38]. Among patients with these associations, Black patients were more likely to exhibit abnormal breast examinations [39].

Physiological and Subclinical Breast Tissue Changes

Subclinical breast tissue changes after treatment were found using biophysical and imaging-based methods. TDC and transepidermal water loss measurements in breast tissue increased significantly post-radiotherapy [48]. Over time, TDC measurements of the breast and lateral thorax decreased [49]. Using US-based phenotyping, three patient clusters were identified, characterized by breast lymphedema prevalence and quality-of-life impact, with those with high prevalence demonstrating a moderate to severe impact [39]. Long-term follow-up studies reported breast edema as part of multiple late treatment-related effects, with severe adverse effects being uncommon [50,51]. In contrast, a cohort with 83.8% of upper extremity lymphedema had more breast symptoms, which correlated significantly with lymphatic dysfunction stage [52].

Discussion

In this review, the current landscape of research that explores the characterization of breast edema and BCRL in the context of BCS was discussed. As the topic of breast cancer and related edema encompasses a vast amount of current research, this systematic review aimed to focus specifically on works that remark on the clinical and demographic contexts of breast edema and BCRL. The various diagnostic methods used to identify and classify breast edema were of particular interest to our review, as there is currently a lack of agreement on diagnostic criteria, which consequently affects research efforts to identify notable correlations. Therefore, we aimed to first establish the different methods of quantitative and qualitative identification of breast cancer-related breast edema before addressing its correlation to BCRL, the latter of which is considerably more well-defined in the literature. We also aimed to identify the current literature that summarizes clinical circumstances associated with an increased likelihood of breast edema, with and without BCRL, as well as factors that increase the likelihood of comorbid expression.

Despite attempts to define breast edema in a more robust, quantifiable manner, multiple methods for diagnosing its presence are still used, including US and TDC measurements, which have been proposed as alternatives to more subjective methods such as self-reported symptom questionnaires. In conjunction with studies that define diagnostic criteria, other studies have focused on applying these criteria to identify clinical risk factors that may increase the likelihood of developing post-BCS breast edema and BCRL. The prevalence of reported breast edema varied widely between studies, likely due to different diagnostic modalities, demographics, sample sizes, and treatment interventions. Nevertheless, breast edema was deemed more likely in the context of axillary lymph node surgery, suggestive of a correlation and possible causation between BCRL and breast edema.

Furthermore, demographic characteristics were noted to be a factor in the risk of developing both breast edema and BCRL; namely, African American race, history of ALND, and occurrence of postoperative complications following BCS were all identified as factors that increase the risk of developing breast edema with comorbid BCRL. Large breast size before BCS and radiation is possibly also linked to an increased risk of edema. Although breast edema and BCRL do not always occur simultaneously, the current literature indicates that the conditions share risk factors and are therefore likely to coexist in some circumstances.

Although the literature on breast edema or BCRL following BCS is vast, very few reviews have addressed the factors and considerations that underlie their coexistence [53,54]. Some reviews focus on the importance of improving the quantification of breast edema [4,55], with complementary reviews on arm lymphedema that address similar diagnostic standards [56]. There is also an extensive literature on breast edema and BCRL in their relationships with BCS, but to our knowledge, no systematic reviews have previously addressed their coexistence in light of shared risk factors.

Limitations of the review process

This study has several limitations that should be acknowledged. First, the search was restricted to English-language publications, which may have excluded relevant papers in other languages. Second, only three electronic databases: OVID, EMBASE, and Web of Science were utilized for this paper. Although these three major databases encompass a large number of papers, other databases may also contain relevant studies. Third, although full-text article screening and critical appraisal of articles were performed by two reviewers using the JBI checklist, they are still subject to reviewer bias in study selection and quality assessment.

Limitations of the Included Studies

The body of included literature demonstrated several important limitations. First, there was substantial heterogeneity in the definition and measurement of breast edema. Studies used varying diagnostic criteria, including clinical assessment, patient-reported symptoms, US-based dermal thickness thresholds (e.g., ≥0.3 mm vs. ≥0.5 mm), and TDC measurements. The lack of consistent standardized outcome measures limits the comprehensive comparability across studies.

Second, sample sizes were widely varied, with some containing small cohorts of n=11 [41], n=24 [48], and n=37 [38], limiting the validity of these outcomes. Third, reporting quality varied across studies, with some failing to clearly specify surgical techniques, radiation protocols, or criteria for breast edema grading. Fourth, most included studies were observational in design and did not report blinding of participants or outcome assessors, increasing the risk of measurement and detection bias.

Gaps in the literature and future research directions

Several gaps were identified in the existing literature. One of the most significant is the absence of a standardized definition and diagnostic criteria for breast edema or breast lymphedema. Studies used heterogeneous assessment methods, including clinical examination, patient-reported symptoms, US-based dermal thickness thresholds, and TDC measurements. Future research should focus on establishing and validating a standardized diagnostic criterion that allows meaningful and accurate comparisons across studies.

Second, although studies were conducted across 14 countries, most were from high-income Western nations. Although the Black race was identified as independently associated with breast lymphedema in two cohorts [33,42], future research should be conducted to determine whether this reflects biological differences, treatment-related factors, or structural and social determinants of health. Future research should also explore racial and ethnic disparities and the underlying mechanisms that may contribute to differential risk and outcomes.

Third, intervention studies remained limited. Although treatment-related factors, such as radiation, nodal irradiation, and chemotherapy, were variably associated with breast edema, a few studies examined preventative strategies or early therapeutic interventions. The potential role of lymphatic sparing surgical techniques, tailored radiation planning, and prophylactic compression therapy needs further investigation. Furthermore, RCTs examining preventative and therapeutic interventions specifically for breast edema are needed.

Fourth, the coexistence and relationship between breast edema and BCRL remain undercharacterized. There are several studies that identified shared risk factors, such as postoperative cellulitis, larger breast volume, and Black race, although a few studies were designed to specifically examine longitudinal progression between the two conditions. Prospective, longitudinal cohort studies are needed to clarify whether breast edema represents an early manifestation of lymphatic dysfunction that predisposes to BCRL, or whether these conditions develop independently but share overlapping pathophysiology.

Implications for practice, policy, and research

The wide variability in the reported prevalence of breast edema emphasizes the need for clinicians to adopt a standardized approach regarding the identification and management of breast edema. Because objective assessment tools, such as US-based dermal thickness measurements and TDC, have consistently shown higher prevalence and subclinical tissue changes than physical examination alone, integrating these image-based modalities with patient-reported symptom measures may improve detection [49]. Early identification and routine surveillance by clinicians can facilitate targeted symptom management of breast edema by enabling timely referrals to lymphedema-focused care and therapies.

Inconsistencies across reported breast edema contribute to its insufficient recognition in clinical practice and limit comparability across studies and healthcare settings. Therefore, policy initiatives should prioritize the development of standardized guidelines for breast edema identification, surveillance, and grading, and their integration into the care of patients with breast cancer. Moreover, the higher chances of breast edema in Black patients reveal disparities across racial groups, highlighting the need for policies that promote equitable access to follow-up care and objective assessments [33,42]. Reimbursement policies that include coverage for diagnostic imaging modalities and long-term follow-up care may improve outcomes for higher-risk patients.

Future studies should focus on standardized measures and diagnostic thresholds for breast edema to improve generalizability. To further understand the physiological processes of breast edema, including its relationship to breast cancer-related arm lymphedema, longitudinal studies are necessary. Recruiting racially diverse populations would help better understand the underlying mechanisms and observed disparities in clinical presentation. Lastly, interventional trials evaluating preventative strategies through objective measures should be prioritized to guide evidence-based clinical management of breast edema.

## Conclusions

This systematic review illustrates that breast edema is a variably reported and under-identified outcome following BCS. The lack of universal assessment methods and follow-up duration strongly affects the reporting prevalence of breast edema. Higher risk is observed in patients with breast cancer with wide-ranging lymphatic dysfunctions, coexisting arm lymphedema, postoperative complications, and a greater impact among Black patients. These findings support the integration of standardized breast edema surveillance and objective assessments into post-surgery breast cancer care to guide future management and prevention strategies.
